# Estimation of the Prevalence of Antimicrobial Resistance in Badgers (*Meles meles*) and Foxes (*Vulpes vulpes*) in Northern Ireland

**DOI:** 10.3389/fmicb.2021.596891

**Published:** 2021-02-18

**Authors:** Maria J. H. O’Hagan, Ana V. Pascual-Linaza, Catherine Couzens, Clare Holmes, Colin Bell, Nessie Spence, Robert J. Huey, Julie A. Murphy, Ryan Devaney, Angela Lahuerta-Marin

**Affiliations:** ^1^Veterinary Epidemiology Unit, Department of Agriculture, Environment and Rural Affairs, Belfast, United Kingdom; ^2^Surveillance and Antimicrobial Resistance Branch, Department of Agriculture, Environment and Rural Affairs, Belfast, United Kingdom; ^3^Bacteriology Branch, Veterinary Sciences Division, Agri-Food and Biosciences Institute, Belfast, United Kingdom; ^4^Disease Surveillance and Investigation Branch, Veterinary Sciences Division, Agri-Food and Biosciences Institute, Belfast, United Kingdom; ^5^Department of Agriculture, Environment and Rural Affairs, Belfast, United Kingdom

**Keywords:** Antimicrobial Resistance, wildlife, ESBL, AmpC, *Salmonella*, MRSA, badgers, foxes

## Abstract

Antimicrobial resistant (AMR) bacteria can be shared between humans and animals, through food, water, and the environment. Wild animals are not only potential reservoirs of AMR, but are also sentinels mirroring the presence of AMR zoonotic bacteria in the environment. In Northern Ireland, little is known about levels of AMR in bacteria in wildlife, thus the current study aimed to estimate the prevalence of AMR bacteria in wildlife using wildlife species from two ongoing surveys as a proxy. Nasopharyngeal swabs and faecal samples from European badgers (*Meles meles*) (146 faecal samples; 118 nasal samples) and red foxes (*Vulpes vulpes*) (321 faecal samples; 279 nasal samples) were collected throughout Northern Ireland and were used to survey for the presence of extended spectrum beta lactamase resistant and AmpC-type beta lactamases *Escherichia coli* (ESBL/AmpC), *Salmonella* spp. (only in badgers) and methicillin resistant *Staphylococcus aureus* (MRSA). ESBLs were detected in 13 out of 146 badger faecal samples (8.90%) and 37 out of 321 of fox faecal samples (11.53%), all of them presenting multi-drug resistance (MDR). Fourteen out of 146 (9.59%) badger faecal samples carried *Salmonella* spp. [*S. Agama* (*n* = 9), *S.* Newport (*n* = 4) and *S. enterica* subsp. *arizonae* (*n* = 1)]. Overall, AMR was found only in the *S. enterica* subsp. *arizonae* isolate (1/14, 7.14%). No MRSA were detected in nasopharyngeal swabs from badgers (*n* = 118) and foxes (*n* = 279). This is the first attempt to explore the prevalence of AMR in the two common wildlife species in Northern Ireland. These findings are important as they can be used as a base line for further research exploring the origin of the found resistance. These results should encourage similar surveys where environmental samples are included to bring better understanding of AMR dynamics, and the impact on wildlife, domestic livestock and humans.

## Introduction

The phenomenon of microbes becoming resistant, due to a generalised inappropriate use of antimicrobials in humans and animals, is currently happening at a global scale for a broad range of microorganisms (Dolejska and Literak, 2019). The direct consequences of Antimicrobial Resistance (AMR) include longer illnesses, increased mortality and increased costs (World Health Organization, 2015).

AMR can occur spontaneously in nature. AMR occurring naturally in some bacterial species is denominated “intrinsic resistance” (OIE, 2012) defined as the innate ability of a bacteria species to resist the action of an antibiotic due to its structural or functional characteristics. However, high levels of AMR are a result of selective pressure exerted on the bacterial population due to the use of antimicrobial agents (Schwarz et al., 2001). Furthermore, as many antibiotics belong to the same class of medicines, resistance to one specific antibiotic agent can lead to resistance to a whole related class. Moreover, resistance that develops in one organism or location can spread rapidly through, for instance, exchange of genetic mobile material such as plasmids between different bacteria (Davies and Davies, 2010). The importance of these AMR bacteria present in humans and animals, which can be transmissible through food, water, and the environment (Arnold et al., 2016a; Dolejska and Literak, 2019), make them a focus point for a “One Health” approach. Regarding wildlife, antimicrobial resistant bacteria occurrence depends on host interaction with potentially anthropogenic impacted habitats by landfills, draining of insufficiently treated wastewaters and wastes from intensively manage livestock farms (Wellington et al., 2013; [Bibr B15][Bibr B53]). However, AMR bacteria in wildlife such as rodents has also been described in areas with low levels of anthropogenic activity (Osterbald et al., 2001). As the continuous exchange of bacteria between environmental niches contributes to their dissemination (Wellington et al., 2013), the role of the environment in relation to AMR is one of the focus areas of the European Union Action Plan (European Commission, 2017).

Little is known about whether AMR is present in the environment and if at all, the levels in zoonotic or commensal bacteria in Northern Ireland. Previous research suggests that the levels of AMR in the British Isles are relatively low compared to other areas such as Africa, parts of South America and Southern Asia and broadly similar to much of continental Europe (Hendriksen et al., 2019). As resistant bacteria are present in wild animals, they can be useful sentinels mirroring the presence of AMR bacteria in an area (Dolejska and Literak, 2019). Wildlife related data can therefore be used to gain a better understanding of the levels of AMR in environmental bacteria (Gilliver et al., 1999; Mo et al., 2018). In the current study, data collected from European badgers (*Meles meles*; order carnivore, omnivorous) (Roper, 1994) and red foxes (*Vulpes vulpes*; order carnivore, omnivorous) (Soe et al., 2017) were therefore used to estimate the prevalence of AMR in these two wildlife species in Northern Ireland.

## Materials and Methods

### Study Population and Design

Wildlife samples were collected as part of two ongoing surveys performed in Northern Ireland. The first of these is a road traffic accident (RTA) survey which is conducted with the aim of estimating the prevalence of bovine tuberculosis, caused by *Mycobacterium bovis*, in European badgers (*Meles meles*) (Courcier et al., 2018). This survey is conducted all year around and has been in place in Northern Ireland since 1998. It involves the collection of up to 350 badger carcasses per year. These carcasses were reported by members of the public and collected by dedicated staff and collection vehicles. Only carcasses deemed suitable for postmortem examination were taken to two veterinary diagnostic laboratories. Detailed procedures for this survey are described previously (Courcier et al., 2018). The second survey aims to establish the geographical epidemiological status of *Echinococcus multilocularis* using red fox (*Vulpes vulpes*) carcasses collected throughout Northern Ireland of which the majority are shot by hunters (for reasons of pest control), while a minority are reported after being killed by a road traffic accident (Courcier et al., 2014). This survey encompasses 325 foxes every year which are tested for *E. multilocularis* in faecal samples in order to demonstrate freedom from this parasite (Commission Delegated Regulation (EU) No 1152/2011 Annex I). Fox carcasses were reported and collected through the same channels as the RTA badger survey involving dedicated reporting systems, collection staff and vehicles. Faecal (badgers, *n* = 146; foxes, *n* = 321) and nasopharyngeal swabs (badgers, *n* = 118; foxes, *n* = 279) were collected from foxes and badgers from September 2018–June 2019. Badgers and foxes were collected within 24–48 h after death in order to prevent autolysis of carcasses. Post mortem procedures were not performed on carcasses in an advanced stage of autolysis.

Samples from badger and fox carcasses were processed for bacteria of interest. These were two Gram negative enterobacteriaceae [Extended spectrum beta lactamase resistant and AmpC-type beta lactamases *Escherichia coli* (ESBL/AmpC), *Salmonella* spp. (only badgers)] and one Gram positive-Methicillin resistant *Staphylococcus aureus* (MRSA).

*E. coli* (ESBL/AmpC) was tested from the faeces of foxes (*n* = 321) and badgers (*n* = 146), *Salmonella* spp. were tested from faeces of badgers (*n* = 146) and MRSA from nasopharyngeal swabs from foxes (*n* = 279) and badgers (*n* = 118). Fox samples were not tested for the presence of *Salmonella* spp. Both fox and badger carcasses were collected throughout Northern Ireland.

### Microbiological Methods

**FIGURE 1:**
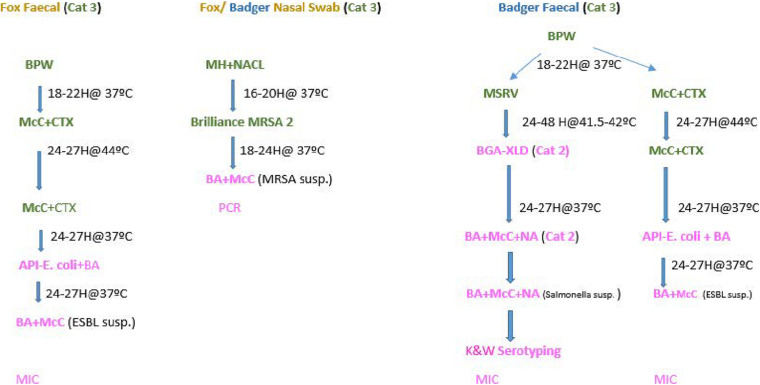
Flow chart- Microbiological processing of faecal and nasopharyngeal swabs collected from foxes and badgers (2018–2019).

The microbiological methods applied in this study are described below and outlined in Figure 1.

#### Microbiological Confirmation and Minimum Inhibitory Concentration (MIC)

It is well documented that badgers can carry *Salmonella* spp. (Wray et al., 1977; Euden, 1990; Wilson et al., 2003). Although *Salmonella* spp. have been occasionally isolated from foxes (Euden, 1990), at present this wildlife species can be consider an incidental host and is still not considered yet a reservoir of *Salmonella* spp. (Chiari et al., 2014). Hence, it was decided, for logistic and budget limitations, that only faecal samples from badgers would be tested for presence or absence of *Salmonella* spp.

Although the three bacteria of interest fall under hazard category two agents and can be processed in a Category two Laboratory (CL2), *M. bovis* and *E. multilocularis* (the agents that the carcasses are primarily collected for), are classified as hazard three agents. Therefore suspected samples must be processed in a Category 3 Laboratory (CL3), due to serious biosafety impact. Thus faecal and nasopharyngeal swabs were initially processed in a CL3. Once any of the microorganisms of interest were isolated, processing/confirmation continued in a CL2 (see Figure 1).

#### ESBL/AmpC

One gram of faeces was inoculated in Buffer Peptone Water (BPW) and incubated at 37°C for 18–22 h. Thereafter, a 10 μl loop was inoculated onto MacConkey agar containing 1 mg/L cefotaxime (CTX) plate. The CTX plates were incubated for 24–27 h at 44°C. Pure colonies from the CTX were then inoculated again on to MacConkey CTX plates to maintain selective pressure and incubated at 37°C for 24 h. Thereafter Analytical Profile Index (API) tests were carried for identification according to manufacturer specifications^1^.

#### Colistin mcr-1

Polymerase Chain Reaction (PCR) was performed according to Liu et al. (2016) on the only colistin resistant *E. coli* detected, to determine the presence of the plasmid-mediated gene mcr-1.

#### *Salmonella* spp.

*Salmonella* spp. were identified and confirmed as described elsewhere (Porter et al., 2020).

### Minimum Inhibitory Concentration (MIC)

Once ESBL and *Salmonella* spp. were identified, individual colonies were set-up in blood and McConkey plates in preparation for MIC. MIC was tested for the recommended set antimicrobials equal for *E. coli* and *Salmonella* spp. specified in Commission Decision EU/652/2013, the MIC technique is described elsewhere (Lahuerta-Marin et al., 2017). The European Committee on Antimicrobial Susceptibility Testing (EUCAST) Epidemiological cut-off values (ECOFFs) were applied as specified in EU Commission Decision EU/652/2013.

The antimicrobials for which the MICs were checked were: ampicillin, azithromycin, ceftazidime, cefepime, cefoxitin, chloramphenicol, ciprofloxacin, colistin, cefotaxime, ertapenam, gentamycin, imipenem, meropenem, nalidixic acid, sulfamethoxazole, temocillin, tetracycline, tigecycline, and trimethoprim. Cefotaxime + clavulanic acid, ceftazidime + clavulanic acid were also included to determine synergy, which allowed to classify them as phenotypic ESBLs and AmpC following growth on CTX media (Figure 1). ESBLs and AmpC were classified based on EUCAST guidelines as follows:

“Presumptive ESBL producers” refers to those isolates with MICs > 1 mg/L for cefotaxime and/or ceftazidime and a synergy test positive for any of these antimicrobials and susceptibility to meropenem (MEM ≤ 0.125 mg/L). These isolates may also harbour other resistance mechanisms (e.g., AmpC-encoding genes).

“Presumptive AmpC producers” refers to isolates with MICs > 1 mg/L for cefotaxime and/or

ceftazidime and cefoxitin MIC > 8 mg/L together with susceptibility to meropenem (MEM ≤ 0.125 mg/L). No distinction between acquired AmpC and natural AmpC was made. These isolates may also harbour other resistance mechanisms (e.g., ESBL-encoding genes).

“Presumptive ESBL + AmpC producers” refers to isolates with the ESBL + AmpC phenotype as described above.

### MRSA

The nasopharyngeal swabs were placed in glass universals containing 10 ml aliquots of Muller Hinton (MH) broth and 6.5% NaCl and incubated for 16–20 h at 37°C. Then a 10 μl loop of the broth was spread on Brilliance 2 MRSA agar and incubated for 24 h at 37°C. Subculture presumptive MRSA colonies were put on to blood agar and incubated for 24 h at 37°C. Presumptive colonies were then confirmed by PCR, according to the protocol for PCR Amplification of mecA, mecC, spa, and pvl validated by the European Reference Laboratory for Antimicrobial Resistance (Eurl-Ar, 2012).

### Statistical Analyses

Data were described and proportions along with 95% confidence intervals (95% CI) were calculated. Percentages resistance and distributions of MIC values were calculated for every antimicrobial. Significant differences of proportions were calculated applying chi-squared tests. All statistical analyses were conducted using R (version 4.0.1; The R Project for Statistical Computing^2^) and maps were produced using ArcMap (version 10.3.1; ESRI).

## Results

### Extended Beta Lactamase and AmpC-Type Beta-Lactamases Escherichia Coli (ESBL/AmpC)

**FIGURE 2:**
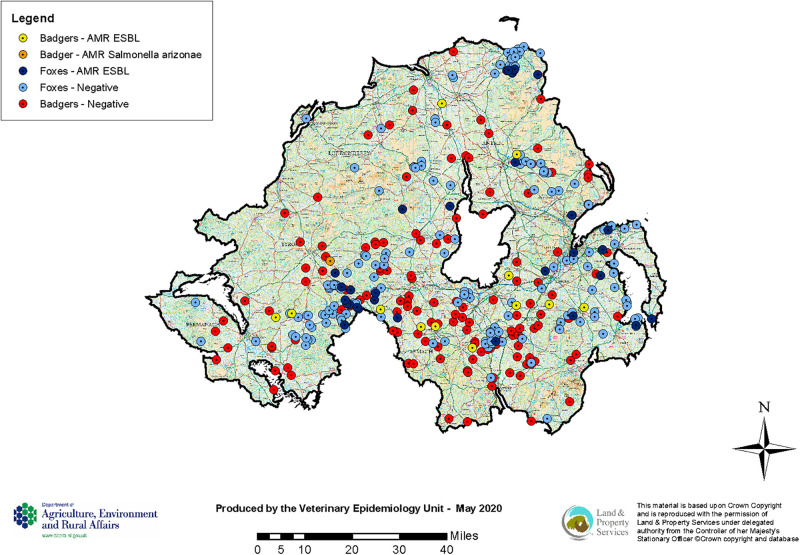
Locations of collected badger and fox carcasses for AMR testing (August 2018–June 2019).

A total of 13 out of 146 badger faeces samples (8.90%; 95% CI 5.02–15.04%) carried ESBL. Fourteen of these isolates showed resistance against any of the tested antimicrobials (Table 1 and Figure 2). ECOFFs were based on European Union guidelines (European Union, 2013).

**TABLE 1:** Distribution of number of samples, isolates, and AMR positive isolates collected from foxes and badgers (2018–2019).

Bacterium	Badgers			Foxes		
	Number of samples tested	Number (%) of isolates	Number (%) of AMR positive	Number of samples tested	Number (%) of isolates	Number (%) of AMR positive
*Escherichia coli* (ESBL/AmpC) (faeces)	146	13 (8.90%)	13 (100%)	321	37 (11.53%)	37 (100%)
*Salmonella* spp. (faeces)	146	14 (9.59%)	1 (7.14%)	0	−	−
Methicillin resistant *Staphylococcus Aureus* (MRSA) (Nasopharyngeal swabs)	118	0 (0.0%)	−	279	0 (0.0%)	−

A total of 37 out of 321 fox faeces samples (11.53%; 95% CI 8.35–15.66%) contained ESBL. All of these 37 isolates showed resistance against any of the tested antimicrobials (Table 1). Tables 2, 3 show the distribution of resistant ESBL/AmpC type *Escherichia coli* found by antimicrobial. Cut off points were based on European Union guidelines (European Union, 2013).

**TABLE 2:** Minimum inhibitory concentrations (MICs) and antimicrobial resistance in ESBL *Escherichia coli* isolated from faecal samples (*n* = 16) from badgers (*Meles meles*) in Northern Ireland in 2018–2019.

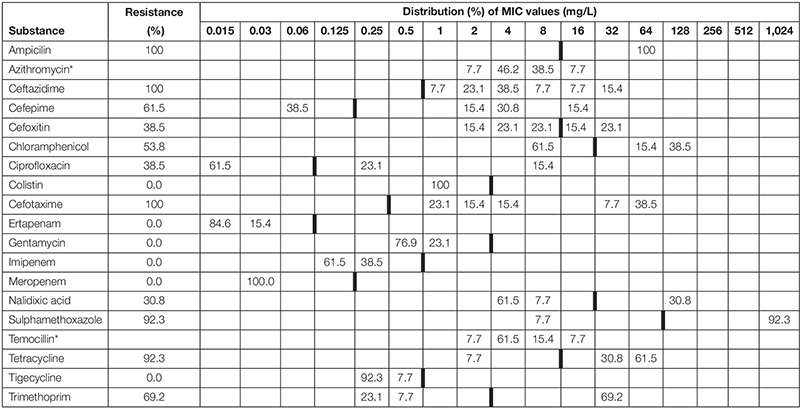

*No EUCAST ECOFF available. Cefotaxime + Clavulanic acid and Ceftazidime + Clavulanic acid were included for MIC, as described in Decision EU/652/2013, to determine if synergy was present or not.

**TABLE 3:** Minimum inhibitory concentrations (MICs) and antimicrobial resistance in ESBL/AmpC isolated from faecal samples (*n* = 39) from red foxes (*Vulpes vulpes*) in Northern Ireland in 2018–2019.

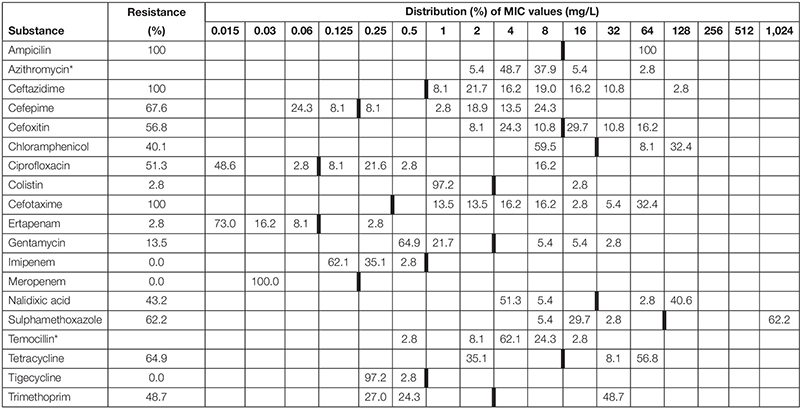

*No EUCAST ECOFF available. Cefotaxime + Clavulanic acid and Ceftazidime + Clavulanic acid were included for MIC, as described in Decision EU/652/2013, to determine if synergy was present or not.

There was no significant difference in the proportion of ESBL/AmpC resistant isolates found in badgers compared to foxes (Chi-squared 0.724, df = 1, *p* = 0.395).

One phenotypic ESBL strain was also resistant to colistin. The strain was negative to *mcr-1*.

### *Salmonella* spp.

*Salmonella* spp. was detected from 14 out of 146 badger faeces samples (9.59%; 95% CI 5.54–15.86%). *S. Agama* was the most prevalent serovar (*n* = 9) followed by *S.* Newport (*n* = 4) and *S. enterica* subsp. *arizonae* (*n* = 1). AMR were only observed in one out of 14 *Salmonella* spp. isolates (7.14%; 95% CI 0.37–35.83%), the *S. enterica* subsp. *arizonae* isolate, which showed resistance against ampicillin (MIC > 64 mg/L), ceftazidime (MIC = 64 mg/L), cefoxitin (MIC > 64 mg/L), cefotaxime (MIC = 32 mg/L), and ertapenam (MIC = 0.12 mg/L) and no synergy with clavulanate (Table 1 and Figure 2). This type of resistance is consistent with AmpC. Overall one of the 146 badger faecal samples collected contained therefore resistant *Salmonella* spp. isolates (0.68%; 95% CI 0.03–4.32).

### Methicillin-Resistant Staphylococcus Aureus (MRSA)

No MRSA was detected in any of the nasopharyngeal swabs of badgers (*n* = 118) or foxes (*n* = 279) (Table 1).

## Discussion

In Northern Ireland, little is known about the prevalence of AMR in wildlife. Therefore, two common wildlife species (European badgers (*Meles meles*) and red foxes (*Vulpes vulpes*)) were used as sentinels to gain insight into this. The current study aimed to survey for the presence of resistance in these two wildlife species in ESBL/AmpC, *Salmonella* spp. (only in badgers) and MRSA.

In the current study 13 out of 146 badger faecal samples contained ESBL with all 13 showing resistance (all against multiple antimicrobials). Furthermore, 37 out of 321 fox faecal samples contained ESBL with all 37 showing resistance (again all against multiple antimicrobials). This is of concern as our results show high correlation between ESBL/AmpC resistance and levels of resistance to other antimicrobials including one isolate resistant to colistin. ESBL is known to be widely distributed in wildlife and is considered to be a key indicator pathogen to trace the evolution of multi-resistant bacteria in the environment and wildlife (Guenther et al., 2011). It was anticipated that ESBL in wildlife would express a multi-resistant phenotype, not due to the nearby use of antimicrobials or antimicrobials in sub-therapeutic concentrations in natural environments, but because distant use had caused a multi-resistant organism to evolve in the first place which subsequently spread to different ecological niches (O’Brien, 2002). The majority of previous research into ESBL prevalence in wildlife has focussed on birds and rodents and prevalence appears to be highest in urbanised areas (Guenther et al., 2011). However, previous research did report the presence of resistant ESBL in foxes (1 out of 7 animals sampled) (Costa et al., 2006) and in badgers (Alonso et al., 2017). It is also documented that plasmids that harbour ESBL and/or pAmpC genes may also carry other resistance genes, meaning that ESBL/pAmpC-producing pathogens can be resistant to other classes of antimicrobial agents as well (MacVane et al., 2014).

*Staphylococcus aureus* is a commensal bacterium with the potential to cause severe disease in humans and animals. MRSA, which is resistant to most β-lactam antibiotics, is a major cause of hospital-associated infections. Livestock-associated (LA)-MRSA has also been recognised to cause infections in humans (Köck et al., 2010) and has been detected in pigs and cattle in Northern Ireland (Hartley et al., 2014; Lahuerta-Marin et al., 2016). The first reported isolation of LA-MRSA in Northern Ireland, and indeed in the UK, was detected in a pig from a mixed swine-dairy cattle herd in 2014 (Hartley et al., 2014). Two on-farm investigations followed and environmental and animal samples were collected. LA-MRSA CC398 t034, the most common strain type in livestock in Northern Ireland (Sharma et al., 2016), was isolated from all environmental samples collected from the first infected farm and from the pig samples only. The bacterium was not detected from any other animal samples (cattle, dog, and sheep) collected form the follow-up epidemiological investigation of the index case (unpublished data). These results showed that LA-MRSA CC398 t034 was restricted to the environment and that the main animal hosts were pigs. However, in the current study no MRSA was observed in nasopharyngeal swabs from either badgers or foxes, hence more research is required and perhaps collection of wildlife around infected farms could be an option for future surveys. It is possible that sampling of badgers and foxes may not be a good proxy or indicator for environmental contamination with this LA-MRSA type. In that case, wildlife may represent an underestimation of levels of LA-MRSA in the environment (if any), thus, the use of wildlife samples as an environmental proxy could be a limitation for this particular pathogen.

Previous studies have demonstrated that a wide range of *Salmonella* spp. is known to be commonly present in badgers in the UK (Taylor, 1968; Wray et al., 1977; Euden, 1990; Wilson et al., 2003). The reported range of serovars is broad with *S. Agama* being the most commonly isolated serovar (Euden, 1990), as observed here. The only resistant serovar *S. enterica* subsp. *arizonae* detected in the current study is one of the less frequently found subspecies of *Salmonella* spp., most commonly detected in reptiles (especially snakes and tortoises) (Hall and Rowe, 1990; Bertrand et al., 2008; Bruce et al., 2018). Strains of *Salmonella* spp. with resistance to antimicrobial drugs are now widespread in both developed and developing countries (Threlfall, 2006). The only resistant isolate in the study was *S. enterica* subsp. *arizonae* showing AMR against five antimicrobials including 3rd generation of cephalosporins. ESBLs *Salmonella* spp. have been detected in chickens (Dierikx et al., 2010; de Souza et al., 2019) and other livestock (Riano et al., 2006). ESBL Salmonella isolations from wildlife are uncommon but ESBL *S. Infantis* isolated from owls have been reported (Fuentes-Castillo et al., 2019). On the other hand, human salmonellosis due to ESBL non-paratyphi *Salmonella* spp. have been described. The UK reported cases of clinical salmonellosis in humans due to ESBL *Salmonella* spp. (EFSA, 2018, 2019). However, at present, ESBL *Salmonella* spp. are uncommon in livestock in the UK, but have been reported by some European Member States (EFSA, 2018, 2019; Uk-Varss, 2019). Genetic characterisation of the strain will provide more clues about the potential origin. In addition, the results of the current study are very interesting as sampled badgers were carrying relatively low levels of *Salmonella* spp., and not many of the isolates carried any AMR compared to AMR levels observed in livestock (Porter et al., 2020). This suggests exposure and dynamics of infection with *Salmonella* spp. in both domestic livestock and wildlife reservoirs such as badgers are different. Nevertheless the impact of carrying ESBL *Salmonella* spp. may be high particularly regarding treatment, as human infections due to *S. enterica* subsp. *arizonae* can occur and have been described before (Gavrilovici et al., 2017; Lakew et al., 2013).

The reservoir of resistance genes in the environment is known to be due to a mix of naturally occurring resistance, those present in animal and human waste and the selective effects of pollutants, which can co-select for mobile genetic elements carrying multiple resistant genes. Resistance genes can be acquired from any source, but gene flow is probably structured by ecology, with species that share similar niches drawing from similar gene pools (Wellington et al., 2013). Several possible transmission routes exist including direct contact with infected individuals, their tissues or their faeces, water and soil (Vittecoq et al., 2016). Once present in the environment, the resistant bacteria can then be potentially acquired by wild animals and then reintroduced to humans. Therefore, the potential of plasmid transfer between strains of the same species or between different bacterial species or genera creates an environmental reservoir of resistance with potentially far reaching impacts for human health (Carroll et al., 2015). As there is a significant level of resistant ESBL/pAmpC- producing pathogens found in wildlife in the current study, similar to previous studies (Costa et al., 2006; Alonso et al., 2017; Darwich et al., 2019), the sources of these would need further investigation focusing on both farm animal and human related origins (Arnold et al., 2016b; Larsson et al., 2018). Potential associations that could be explored include water sources, human and farm animal population density, and proximity to hospitals and pharmaceutical industries (Arnold et al., 2016b). Further molecular characterisation into ESBL and *Salmonella* spp. would also be useful in order to provide insight into the possible correlation between phenotypic and genotypic patterns of resistance between isolates from wildlife, livestock and humans. The results of further research could help prioritising the development of effective One Health strategies to mitigate the spread of AMR into the environment in targeted areas in Northern Ireland, such as: pre-treatment of manure before use as fertiliser; pre-treatment of waste across the farm to slaughterhouse continuum before discharge into the general sewage system; education to increase awareness of all hospital personnel on hygiene, sanitation and safe disposal practices; insurance of the safe disposal of antimicrobial medicines and hazardous waste; consideration of pre-treatment of hospital waste before discharge into the general sewage system (FAO, 2018). Furthermore, research into the possible correlation of AMR bacteria in wildlife/livestock and their environment would be useful, because monitoring of AMR in wildlife and the environment can be used as an early detection of new AMRs (Martínez, 2009; Radhouani et al., 2014).

This survey is the first step to assess the risk of wildlife as a potential environmental reservoir of antimicrobial resistance for domestic livestock and humans. We expect that these results encourage the Environmental Agency in Northern Ireland to test bacteria isolated from environmental samples- soil, dust and water. Hence this will contribute to a better one health understanding of AMR (European Commission, 2017).

## Limitations of the Study

Only one ESBL strain also presented resistance to colistin. It was previously described that plasmid-mediated *mcr-1* had resistance to this critically important antibiotic (Liu et al., 2016). This PCR was performed as an initial screening. Since then, 10 plasmids have been described (*mcr-1–10*). There is no validated PCR available to detect presence or absence of all 10 *mcr*s. Further analyses will be conducted in relation to testing for *mcr-1*. In the near future, there are plans to test for *mcr-1–5* following the validated PCR protocol developed by EURL-AR^3^. If the colistin resistant ESBL carry any of *mcr-6–10* plasmids, we will be able to detect it when we perform whole genome sequencing on this isolate. This was not performed in this initial stage of this survey.

No microbiological isolation of commensal *E. coli* was performed for this study. If the survey is performed again in the near future, it would be desirable to calculate the prevalence of commensal *E. coli* and the levels of resistance of this bacterium carried by wildlife species in Northern Ireland.

Due to the nature of the sampling within the surveys (convenience sampling) and the relatively low numbers of positive samples involved, it is not possible to identify clusters and potentially relate them to features such as rivers or drainage basins. Further research could be conducted to address this.

Wildlife sampling for AMR is important (based on the “One Health” concept), but challenging. Similar to other research (Mo et al., 2018), dead animals were therefore the sample source for this study. This provides difficulties in relation to autolysis. However, in the current study this was prevented as much as possible by collecting and processing the badgers and foxes within 24–48 h after death.

This project has provided the first insight into the prevalence of AMR in wildlife as a proxy for the environment. Results showed that ESBL/AmpC were the most prevalent type of resistance in badgers and foxes. Moreover, the ESBL/AmpC isolates recovered were also resistant to several other antimicrobial agents. AMR levels in *Salmonella* spp. were very low but highly resistant. The resistance pattern was unusual in that resistance against for example, AmpC and MRSA was not detected. These results suggest that the three pathogens may have different dynamics of infection and exposure from the environment into the sampled wildlife species. These findings will form a baseline for further research and are an important first step in our understanding of the levels of AMR bacteria in wildlife and potentially the environment.

## Data Availability Statement

The original contributions presented in the study are included in the article/Supplementary Material, further inquiries can be directed to the corresponding author/s.

## Ethics Statement

Ethical review and approval was not required for this study because the animals were already deceased. Animal carcasses (badgers and foxes) were collected from the roadside after becoming victims of road traffic accidents or, in the case of some of the foxes, were shot by hunters (not for the purpose of the study).

## Author Contributions

AP-L and AL-M conceived and designed the study. MO’H and AL-M wrote the manuscript, with interpretation of results and discussion inputs from AP-L. JM organised collection of the samples. CC, CH, CB, NS, and RD performed laboratory work. MO’H carried out statistical analyses. RH approved the concept of the study and liaised to initiate the study. All authors read and approved the final manuscript.

## Conflict of Interest

The authors declare that the research was conducted in the absence of any commercial or financial relationships that could be construed as a potential conflict of interest.

## Footnotes

1 https://www.biomerieux-usa.com/clinical/api

2 https://www.r-project.org/

3 https://www.eurl-ar.eu/CustomerData/Files/Folders/21-protocols/396_mcr-multiplex-pcr-protocol-v3-feb18.pdf
